# Septic Shock From Pan-Spinal Epidural Abscess Attributed to Recent Acupuncture and Trigger Point Injections for Acute Lower Back Pain in a Previously Undiagnosed Diabetic Patient: A Case Report

**DOI:** 10.7759/cureus.40088

**Published:** 2023-06-07

**Authors:** Shaheryar Usman, Faraz Badar, Carlos Collado, Andrew Weber, Alan Kaell

**Affiliations:** 1 Internal Medicine, Mather Hospital, Northwell Health, Port Jefferson, USA; 2 Critical Care and Pulmonary Medicine, Mather Hospital, Northwell Health, Port Jefferson, USA; 3 Critical Care and Pulmonary Medicine, Donald and Barbara Zucker School of Medicine at Hofstra/Northwell, Hempstead, USA

**Keywords:** trigger point injections, immunocompromise, acupuncture, imaging, dka, diabetes, case report, shock, spinal, epidural abscess

## Abstract

Epidural abscesses can lead to devastating neurological consequences if not diagnosed and managed in a timely manner, especially in immunocompromised patients. We report the case of a 60-year-old woman with undiagnosed diabetes mellitus who presented to the hospital with a complaint of progressive altered mental status for the past two days. Eight days prior to presentation, the patient tripped over a pillow at home and developed mildly nagging, acute lower back pain. Upon the recommendation of her friends, she underwent two sessions of acupuncture around the lumbar area on days six and five prior to being brought to the hospital. She also saw her primary care physician on day three prior to presentation, who performed a history and physical examination and, after feeling that she did not have any red flags, empirically administered lidocaine-based trigger point injections near the same lumbar areas with the patient's consent. On the day of presentation, the patient fell at home and was unable to walk, after which she was immediately brought to the hospital, where she demonstrated toxic metabolic encephalopathy due to diabetic ketoacidosis (DKA) and lower extremity paraplegia. Emergent imaging revealed a pan-spinal epidural abscess (PSEA) after an attempted lumbar puncture led to immediate pus in the syringe.

Diagnosing an epidural abscess can be difficult as signs and symptoms can mimic other conditions such as meningitis, encephalitis, and stroke. High suspicion on the physician’s end is needed when a patient presents with acute back pain, fevers, and neurological deterioration if the condition is otherwise unexplained, and especially in the presence of risk factors for PSEA that may be recognized only upon presentation.

## Introduction

Acupuncture is a form of complementary and alternative medicine that can help treat medical conditions including chronic lower back pain, cancer-associated pain [1], nausea and vomiting, headaches, Parkinson’s disease [2], and augment smoking cessation success. Acupuncture, in general, has a good safety profile; however, several cases of adverse outcomes, including spinal epidural abscess (SEA), have been reported [3]. Risk factors for adverse outcomes include diabetes mellitus, alcoholism, and underlying malignancies or immunosuppression. We present a rare case of pan-spinal epidural abscess (PSEA) with rapidly progressive lower extremity paraplegia status post-acupuncture and trigger point injections around lumbar areas in the setting of newly diagnosed diabetes mellitus complicated by diabetic ketoacidosis (DKA) upon admission.

## Case presentation

A 60-year-old woman with a history of hypertension, gestational diabetes mellitus (approximately 30 years ago), colon cancer status post hemicolectomy approximately 10 years ago (currently in remission), and recurrent urinary tract infections (completed a course of Macrobid four days prior to admission) was brought  to the emergency department (ED) with progressive confusion for the past two days. To the best of the patient’s husband’s recollection, she tripped over a pillow at home eight days prior and developed mild acute lower back pain. Upon the recommendation of her friends, she underwent two sessions of acupuncture (her husband was present during the procedure) around the lumbar area on days six and five prior to being brought to the hospital. She also saw her primary care physician (PCP) on day three prior to presentation, who performed a history and physical examination and, after feeling that she did not have any red flags, empirically administered lidocaine-based trigger point injections near the same lumbar areas with the patient's consent. During the procedure, the PCP wore gloves and cleaned the area with an alcohol swab. On the day of presentation, the patient fell at home and was unable to walk, after which she was immediately brought to the hospital. In the emergency room, the patient had a temperature of 102°F, an increased heart rate of 129 beats per minute (b), and a mean arterial pressure (MAP) of 55-60 mmHg. She was confused and intermittently arousable on the initial evaluation. Neurological examination revealed bilateral lower extremity paraplegia and bilateral upper extremity paresis without any sensory deficit. Laboratory investigation revealed a non-compensated high anion gap metabolic acidosis (anion gap of 32) secondary to diabetic ketoacidosis (DKA) with HbA1c of 11.5% and lactic acidosis. Further admission labs are summarized in Tables 1-3.

**Table 1:** Pertinent blood work results

Lab values	Results	Reference range
White blood count	13,000	4,500-11,000 mg/dL
C- reactive protein	>350	< 10.0 mg/L
Erythrocyte sedimentation rate	76	≤ 20 mm/hr
Lactate	11.2	0.5 to 2.2 mmol/L
Procalcitonin	13.6	<0.05 ug/L
Sodium	138	135-145 mmol/L
Potassium	1.9	3.5 to 5 mmol/L
Chloride	94	96 to 106 mmol/L
Bicarbonate	12	24 mmol/L
Blood urea nitrogen	29	<20 mg/dl
Creatinine	1.26	0.7-1.2 mg/dL
Glucose	>400	<140 mg/dL
Hemoglobin A1C (HbA1C)	11.50%	<5.7%

**Table 2:** Urine analysis results

Test	Results	Reference range
Glucose	>1000 mg/dl	Negative
Ketones	40 mg/dl	Negative
Blood	Positive	Negative
Protein	100 mg/dl	Negative
Leukocyte esterase	Trace	Negative
White blood cells (WBC)	3-5 HPF	0-5 HPF
Red blood cells (RBC)	6-10 HPF	0-4 HPF
Bacterial routine urinalysis	Few	Negative

**Table 3:** Arterial blood gas (ABG) was obtained 15-20 minutes after the patient received IV fluid resuscitation and electrolyte replenishment. Blood work showed non-compensated high anion gap metabolic acidosis (anion gap of 32). pH: acid-base balance of the blood; PCO2: partial pressure of carbon dioxide in arterial blood; pO2: partial pressure of oxygen; HCO3: concentration of bicarbonate in arterial blood; O2: oxygen; NRB: nonrebreather

Test	Results	Reference range
pH arterial	7.31	7.35- 7.45
pC02 arterial	41	35- 45 mmHg
p02 arterial	234	80 -110 mmHg
HC03 arterial	20.6	21-28 mmol/L
02 sat% on 100% NRB	99.60%	95-99%

She was started on a norepinephrine drip via peripheral vascular access for pressure support and intubated for airway protection immediately to avoid aspiration. Broad-spectrum antibiotics, including Zosyn IV and vancomycin IV, were initiated.

**Figure 1:**
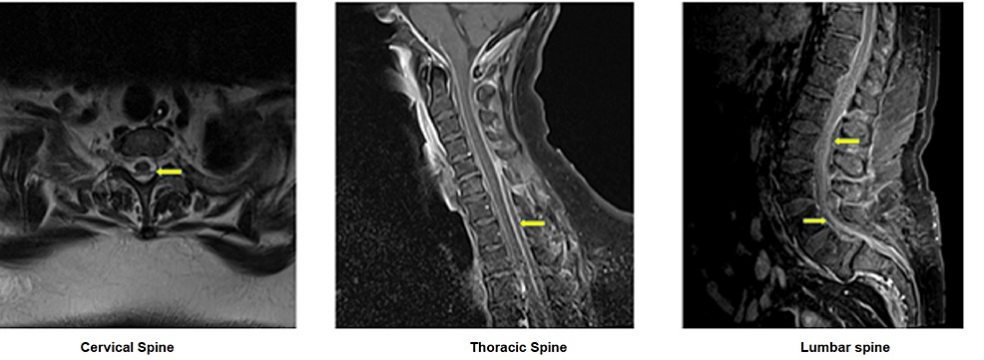
Impression of the cervical, thoracic, and lumbar spine MRI with contrast; yellow arrows show epidural abscess collections around the cervical, thoracic, and lumbar areas, respectively.

Radiographically, a computed tomography (CT) scan of the head, chest, abdomen, and pelvis did not reveal any pathology. A CT scan of the cervical, thoracic, and lumbar spine with IV contrast showed multilevel bilateral thoracic and lumbar nerve root impingements. Due to high suspicion of meningitis, a lumbar puncture was attempted but then aborted immediately due to a grossly purulent aspirate. The patient then underwent urgent magnetic resonance imaging (MRI) of the whole spine with and without gadolinium, which revealed an epidural abscess extending from C1 through the sacrum with paraspinal soft tissue involvement at the T6-T8 and L3-L5 regions and circumferential compression of the spinal cord in the thoracic spine secondary to mechanical obstruction by the abscess (Figure 1).

Neurosurgery was immediately consulted, and the patient underwent emergent cervical, thoracic, and lumbar laminectomies with the evacuation of a multi-level spinal epidural abscess. Postoperatively, the patient was admitted to the intensive care unit (ICU) and was placed on broad-spectrum IV antibiotics. Twenty-four hours later, initial blood and cerebrospinal fluid (CSF) cultures revealed methicillin-sensitive *Staphylococcus aureus* (MSSA) sensitive to cefazolin. Antibiotics were then narrowed to cefazolin IV. The patient underwent transthoracic echocardiography (TTE) followed by transesophageal echocardiography (TEE), which were both negative for bacterial endocarditis. Over the next two weeks, the patient’s mental status continued to progressively improve, and bilateral upper extremity paresis resolved. However, the bilateral lower extremity paraplegia persisted. She was successfully extubated on hospital day six. Repeated blood and urine cultures continued to be negative.

On hospital day 17, the patient's abdomen progressively became distended with diminished bowel sounds, followed by rapid hemodynamic, respiratory, and metabolic instability. The patient again required intravenous vasopressors for vasoplegic shock and underwent repeat endotracheal intubation with invasive mechanical ventilation a second time during the hospital stay to prevent aspiration pneumonia. An urgent CT scan of the abdomen without contrast demonstrated a large burden of pneumoperitoneum in the setting of possible underlying bowel ischemia. General surgery was immediately consulted, and the patient underwent an exploratory laparotomy with a right hemicolectomy and the creation of an end ileostomy due to feculent peritonitis secondary to cecal perforation. Postoperatively, the patient was re-initiated empirically on broad-spectrum vancomycin and meropenem IV. Repeat blood cultures returned positive for MSSA again, and cefazolin IV was reinitiated. The patient was closely monitored thereafter in the ICU. She was extubated for the second time on hospital day 26. Her overall condition improved clinically, apart from persistent bilateral lower extremity paraplegia. Repeat brain and spine imaging did not show any cord compression.

On hospital day 32, a peripherally inserted central catheter (PICC) was placed, and she was discharged to subacute rehab with the plan to complete a 10-week course of cefazolin IV as per infectious disease recommendations along with intense physical therapy. The patient was to follow up with a neurologist and neurosurgeon for a serial MRI of the spine for further evaluation.

As of 12 months post-discharge, the patient has regained only minimal motor strength in both feet, with the ability to flicker some of her toes bilaterally. Recovery has been complicated by the development of extensive stage three to four decubitus ulcers in the sacrum, coccyx, bilateral buttocks, and thighs, requiring multiple hospitalizations and debridement.

## Discussion

A spinal epidural abscess is a bacterial infection that occurs between the vertebral body and the dura mater of the spine. Infection gains access to the epidural space by primarily three routes of access: contiguous spread from a surrounding infected musculoskeletal structure, hematogenous spread from a remote source, or iatrogenic [2] causes, including spinal instrumentation such as the placement of an epidural catheter for anesthesia induction or steroid injection for analgesia [4]. Risk factors augment an individual’s likelihood of contracting an infection or immunosuppression.

Diabetes has been found to be an important risk factor for SEA formation. Other important risk factors for SEA include HIV infection, osteomyelitis, alcoholism, tattooing, and acupuncture [5]. In our patient, although she did not have a diagnosis of diabetes mellitus, historically, she had carried out a diagnosis of gestational diabetes for approximately 30 years. Most likely, she surreptitiously developed adult-onset diabetes over time, which led to impairment of her immune system, making her susceptible to SEA. Multidisciplinary discussion among the emergency medicine, internal medicine, neurosurgical, neurological, and infectious disease experts did not think her acute low back pain following her tripping over a pillow was the manifestation of SEA, although, without imaging, we concede the limitation of our case report is evident but unlikely.

Acupuncture and trigger point injections, if done with improper or non-aseptic techniques or by an inexperienced practitioner, can lead to the introduction of the pathogen(s) either directly into the epidural space or into an adjacent structure that can access the epidural space [6-7]. Although there are no nationally recommended guidelines in the USA for acupuncture and trigger point injections, some states have chalked out certain recommendations, including the use of pre-sterilized disposable needles, functioning sterilization equipment for sanitizing non-needle equipment used for the treatment of patients, handwashing and wearing sterile gloves, and the use of face masks prior to performing the procedures. The patient’s husband mentioned that he was present at the time when she underwent acupuncture and endorsed that needles were taken out of clean packaging and were not reused. During trigger point injections, the physician used gloves and cleaned the areas of injection with alcohol. Although we cannot rule out entirely that SEA was not brewing previously, we suspect that acupuncture caused the seeding of MSSA bacteria into paraspinal space, from where bacteria gained access to epidural space in the setting of poorly controlled diabetes. *Staphylococcus aureus* is found to cause about two-thirds of cases of SEA [8-9]. Other microorganisms include gram-negative bacilli, *Streptococci*, coagulase-negative *Staphylococci*, and anaerobes. Fungi and parasites are rare etiologic causes.

There are certain "red flags" related to acute back pain (including historical and physical markers) that guide physicians to evaluate for potentially dangerous underlying conditions, requiring further investigations. Some of these red flags include trauma, age less than 18 or more than 50, fevers, unexplained weight loss, neurological symptoms, atypical back pain (at night or lying down), and a history of cancer or IV drug use [5,10-12]. The identification of red flags warrants close attention and further diagnostic testing. Our patient had at least two red flags, including age greater than 50 years and a history of colon cancer, but she did not undergo any imaging prior to ED presentation, likely because her complaints were of mild, nagging, acute lower back pain after tripping at home.

MRI with gadolinium-enhanced contrast is considered the imaging test of choice for diagnosing spinal epidural abscesses. MRI can identify the location, degree of inflammation, and separation of epidural soft tissue edema from epidural abscess at an early stage of the disease. If MRI is not available, a CT myelogram would be the second-best imaging modality. Pre-test probabilities of making a proper diagnosis can be enhanced when there is a patient with otherwise unexplained fevers, neurological deficits, and/or encephalopathy who carries appropriate risk factors [13].

Despite the appropriate intervention, the prognosis can vary. Irreversible paraplegia is one of the gravest complications of the spinal epidural abscess [14-15]. Neurological recovery after the onset of paraplegia or quadriplegia due to a spinal epidural abscess varies significantly. The duration of the neurological deficit prior to admission and/or surgical intervention may have a large impact on the recovery pattern [16] and is unlikely to reverse if neurological symptoms last for more than 24-36 hours [4]. As per previous retrospective analyses [17-18], 5%-7% of patients with SEA die due to uncontrolled sepsis or other complications, and 4%-22% of patients develop irreversible paraplegia. In our case, MRI with contrast was diagnostic and allowed rapid diagnosis and appropriate neurosurgical intervention, which likely prevented further progression of neurological deficits (improved bilateral upper extremity paraparesis post-neurosurgical intervention) and mortality.

## Conclusions

As the popularity of acupuncture and trigger point injections increases, physicians should be aware of their rare but potentially serious complications, including SEA. Primary care physicians should monitor such patients closely after the procedures and have a low threshold for further imaging or hospital evaluation if patients develop even minimal neurological symptoms. Although there are no national-level guidelines with respect to aseptic protocols for acupuncture and trigger point injections in the USA, a review of the literature and various state laws recommends the use of pre-sterilized disposable needles, functioning sterilization equipment for sanitizing non-needle equipment used for the treatment of patients, handwashing, wearing sterile gloves, and using face masks prior to performing the procedures. We emphasize the importance of timely recognition of SEA when presentation includes fevers, encephalopathy, and neurological deficits with appropriate risk factors like diabetes and any spinal intervention, including acupuncture and trigger point injections in the back. We also recommend timely imaging and neurosurgical evaluation within 24 to 36 hours of the onset of paralysis in an attempt to reverse or prevent further neurological deficits and mortality.
